# Balneology-Geologic Considerations

**Published:** 1917-01

**Authors:** Felix Von Oefele

**Affiliations:** New York


### Balneology-Geologic Considerations.

Dr. FELIX VON OEFELE, New York.

In the palaeozoic era the New England moutains and the Appalachian Mountains were located on the immediate eastern seashore; this is to say that this old seashore of the Middle and South Atlantic areas was further west of the recent seashore. The mesozoic seashore was further east in New England, and, in the South Atlantic area, further west. The kaenozoic motion was an increased emersion in the south and a submersion of the plains of the shore in the north. The original cuneiform shape of the combined Atlantic areas changed to a broad ribbon shape. Very far north of the Canadian Shield, the Hudson Bay submerged, and emerged in the palaeozoic era, and submerged again in the sea in the kaenozoic era. The eastern coast plains of mesozoic and kaenozoic group of New England deposits are entirely lost.

The intrusive rocks of the areas are important for the naturally yielded magmatic waters. The relationship of elevation of mountain systems and volcanic eruptions and intrusions allow for the classification of magmatic waters together with the stratum springs in the same division of areas. A large fault filled by intrusive rocks has been caused in precambric era as the central axis of the elevation of the Adirondack-Appalachian chain. It forms the most eastern district of the Eastern Mississippi area. A bifurcating branch splits and goes in the South Atlantic area where it borders on the Middle Atlantic area, and is the cause of the irregular arrangement of the South Atlantic area. In the mesozoic era a parallel broad intrusion happened toward the east, but is interrupted in its continuity in the Middle Atlantic area. It is evident in the South Atlantic area and in New England. The Pacific shore of Canada, then again Sierra Nevada and Baja California form a large intrusion of Post-Cambrian eras. An intrusive island of the same era is the Salmon Mountains. The kaenozoic era formed many irregular intrusions east of the Pacific Post-Cambrian intrusions, partly reaching the Rocky Mountains. The intrusions of different eras show different petrographic character with influence upon the yielded local mineral springs.

We will learn of large fields or fault lines with a large number of similar neighboring springs. The analytical chemical differences are very little. This circumstance has handicapped the profitable development. The large metropoles, whence come most of the sick people visiting spring resorts, have only some springs of very few classes in the neighborhood, lacking the kinds mostly needed.

### SUB-DIVISION INTO BALNEOLOGICAL DISTRICTS

The areas mentioned are based on the orographical character of North America. Their single districts will be based on geology with regard to systems of strata and intrusions.

The relationship between the stratigraphic condition of the surface and the percentage of mineralisation of the spring water has already been shown in the Federal research on the Iowa waters published in 1912. There, generally, the rule is learned that the more recent strata deliver a higher percentage of total solids to the water than the older strata.

These increases show some exceptions. The petrographical influence may be reversed to the geological period. Limestones or dolomites deliver a higher percentage, and sandstones and silicates a lower than expected by this rule. The Ordovician districts of the state of Iowa show 0.02 to 0.04% total solids, Silurian 0.03 to 0.05%, and Devonian 0.03 to 0.10% dissolved in average waters.

The regular arrangement of many geological districts leads to a certain regular arrangement of characteristic waters within the special area. The total solids of Iowan mineral water increase from northwest to southeast. Then, too, the respective figures increase from a covering Iowan to a Kansan, and farther to an Illinoian drift. Wells with less than 0.05% total solids are found within the lower Carbonian in the northwestern part of the state. The total solids of the wells of the later Carbonian are 0.2 to 0.5% in the south and especially in the southwest. The northwestern part of Iowa contains cretaceous deposits with wells of 0.12 to 0.24% total solids.

The influence of geological conditions has been recognized also for the mineral springs of Saratoga, N. Y. John M. Clarke is the geologist of the State of New York. He has the best intentions toward Saratoga Springs as the state property. In 1910 he ordered a re-collection and revision of all evidences concerning the geological surface. He also expects to be able to make additional thrillings for research there.

The decline area of the Rocky Mountains, the Mountain area and the Pacific area are of recent geological date influenced by recent volcanic action. The other five areas belong closer together. That will be seen from the following subdivision into balneological districts. A balneological division and subdivision of North America into systematical areas and districts has never been done hitherto. This first trial cannot be expected to be perfect. The balneology of the future has to correct and to improve my work. I am not a friend of cowardly watchful waiting, and have never been afraid to make some errors approaching further perfection and to become corrected. The coward may evade these errors. I prefer being an errant fighter for research to being a lazy coward.

A preliminary subdivision of the United States subject to corrections by complete study may follow.

I. MIDDLE ATLANTIC AREA

1. Pleistokaenic district of marine deposits,
2. District of Meiokaen and moraines,
3. Oligokaenic district,
4. Cretaceous district,
5. Triassic dictrict with jurassic volcanic intrusions,
6. Palaeozoic district including the Catskill Mountains,
7. Proterozoic district.

II. SOUTH ATLANTIC AREA.

1. Marine Oligokaenic district,
2. Cretaceous district,
3. Precambric rocks with mesozoic intrusions,
4. Climatic resorts of Florida.

III. NEW ENGLAND AREA.

1. Nitrated and Borated faults,
2. Silicated low mineralized springs,
3. Sodium chloride spring of the seashore.

The principal districts of New England have already been published in the Buffalo Medical Journal.

IV. EASTERN MISSISSIPPI AREA.

This area will be especially interesting for the readers of the Buffalo Medical Journal.

1. Cambrian and Ordovician ribbon, including a small western part of Newfoundland, a small ribbon on the south shore of St. Lawrence River and the northwest corner of Vermont.

2. Tahonic Mountains and Saratoga fault,

3. Silurian and Devonian of the upper state of New York and vicinity. This district includes the city of Buffalo.

4. Blue Ridge of the Appalachian system including precambric intrusions and Cambrian and Ordovician deposits,

5. Silurian and Devonian western decline of Appalachian Mountains,

6. Later palaeozoic massive of western Pennsylvania, Ohio, Virginia, Kentucky, Tennessee and Alabama,

7. Silurian and Devonian massive of western Ohio, Indiana and Kentucky,

8. Later palaeozoic district of Michigan,

9. Later palaeozoic district of Illinois, Indiana, Kentucky and Tennessee.

### V. WESTERN (AND SOUTHERN) MISSISSIPPI AREA.

1. Kaenozoic and Mesozoic district of western Tennessee, southern Alabama, Mississippi and Louisiana,
2. Kaenozoic seashore of Texas,
3. Later Tertiar of Nebraska, Colorado, Kansas, New Mexico and northwestern Texas,
4. Continental Kaenozoic deposits of North Dakota, Montana and Wyoming,
5. Cretaceous district of Texas overleaping to Mexico,
6. Cretaceous district of western Minnesota, western Iowa, North Dakota, South Dakota, eastern Nebraska and a small part of Kansas,
7. Later palaeozoic district of southern Iowa, western Arkansas, Oklahoma and northwestern Texas,
8. Silurian and Devonian district of eastern Iowa,
9. Presiluric district of Ozarks,
10. Presiluric district of Wisconsin.

### VI. AREA OF ROCKY MOUNTAINS DECLINE.

1. Eastern spots of old rocks of North Dakota, Minnesota and Sioux Falls,
2. Precambric Black Hills of South Dakota,
3. Precambric Bighorn Mountains,
4. Precambric Front Range,
5. Precambric Sangre de Christo Mountains,
6. Mesozoic deposits east of the Front Range,
7. Southern part of Rocky Mountains of carboniferous system.
8. Kaenozoic continental deposits of western Wyoming and Utah with Uinta Mountains,
9. Mesozoic deposits of Arizona and New Mexico with small mesozoic intrusions,
10. Field of Kaenozoic intrusion of San Juan Mountains,
11. Field of Kaenozoic intrusion on the boundary of Arizona and New Mexico,
12. Large field of Kaenozoic intrusions of the west shore of Mexico.

### VII. MOUNTAIN AREA.

1. Old palaeozoic Salmon River Mountains with palaeozoic and mesozoic intrusions,
2. The very much mixed district of western Utah and Nevada,
3. Palaeozoic district of northwestern California,
4. Large field of kaenozoic intrusions between 45 and 46° lat.; the Blue Mountains of Columbia and Oregon,
5. The Pi-shape connecting Cascade Mountains between the previous and the next district,

6. Large district of kaenozoic intrusions from Yellowstone Park to Cape Mendocino,

7. District of Salt Lake and West Utah and Nevada, with very many intersections of very old and very young deposits,

8. District of intersected kaenozoic intrusions of Southern Nevada,

9. Kaenozoic deposits of southern California and southern Arizona,

10. Old intrusions of Sierra Nevada,

11. Old intrusions of Baja California.

VIII. PACIFIC AREA.

1. Kaenozoic shore of Columbia and Oregon,
2. Kaenozoic shore of southern California,
3. Mesozoic shore of middle California,
4. Palaeozoic shore of northern California.

---

**Autotherapy in Ivy Poisoning.** Chas H. Duncan,, N. Y., N. Y. Med. Jour., Nov. 4. The patient was severely poisoned during defecation in the woods over a clump of rhus, species not determined. (A similar case attracted attention in Buffalo, many years ago, from the same general cause with direct application of the leaves, in lieu of newspaper, toilet paper not then being on the market.—Ed.) In order to secure absolutely specific antibodies, the patient was directed to return to the same place, select a leaf from the same plant (let us trust that the localization was not too exact), chew it and swallow the juice. Within three days he was able to resume his duties as butler. (Dr. Duncan is a graduate of the N. Y. Homoeopathic College, but it should be noted that he advocates autotherapy on broad theoretic and empiric grounds, and that even the insistence on the particular plant is not based on the conception of using a "hair from the dog that bit you," but on the scientific superiority of specific antibodies. We are not yet prepared to accept autotherapy in toto, but it must be admitted that many reports, especially from the war zone, support it to a surprising degree. The licking of wounds is actually prescribed and with claims of good results. If autotherapy is established, even with limitations, it will be only fair to credit Homoeopathy with a far-sighted if somewhat vague conception, of an important therapeutic principle).

---

**Sublingual Medication.** W. Paulson, Practitioner, Oct., advocates this method, not merely in an emergency when the hypodermic syringe is not available but as quicker, safer, cleaner and more reliable—of course mainly for alkaloids.

